# The methanol extract of *Guettarda speciosa* Linn. Ameliorates acute lung injury in mice

**DOI:** 10.1186/s12906-020-2828-6

**Published:** 2020-02-07

**Authors:** Kyun Ha Kim, Ji Yeon Lee, Seonju Ahn, Ran Won, Sang-Jun Kim, Seung-Il Jeong, Jung Ju Lee, Jong-In Kim, Jun-Yong Choi, Myungsoo Joo

**Affiliations:** 1 School of Korean Medicine, Pusan National University, Yangsan, 50612 Republic of Korea; 2 Department ofs Biomedical Laboratory Science, Division of Health Sciences, Dongseo University, Busan, 47011 Republic of Korea; 3 Jeonju AgroBio-Materials Institute, Jeonju, 57810 Republic of Korea; 4 Department of Acupuncture and Moxibustion, College of Korean Medicine, Kyung Hee University, Seoul, 02447 Republic of Korea; 5 Department of Internal Medicine, Korean Medicine Hospital of Pusan National University, Yangsan, 50612 Republic of Korea

**Keywords:** *Guettarda speciosa* Linn., Acute lung injury, Anti-inflammation, Neutrophil elastase, Nrf2, NF-κB

## Abstract

**Background:**

*Guettarda speciosa* is mainly found in tropical areas in Asia. Although *G. speciosa* is traditionally used to treat some of the inflammatory disorders, the experimental evidence supporting the anti-inflammatory effect of *G. speciosa* is limited. Here, we sought to obtain evidence that *G. speciosa* has anti-inflammatory activity using an acute lung injury (ALI) mouse model and to explore possible underlying mechanisms for the activity.

**Methods:**

The methanol extract of *G. speciosa* Linn. (MGS) was fingerprinted by HPLC. Cytotoxicity was determined by MTT and flow cytometer. As for an ALI mouse model, C57BL/6 mice received an intratracheal (i.t.) injection of lipopolysaccharide (LPS). The effects of MGS on lung inflammation in the ALI mice were assessed by differential cell counting and FACS of inflammatory cells and hematoxylin and eosin staining of lung tissue. Proteins were analyzed by immunoprecipitation and immunoblotting, and gene expression was by real-time qPCR. Neutrophil elastase activity was measured by ELISA.

**Results:**

MGS did not cause metabolic disarray or produce reactive oxygen species that could induce cytotoxicity. Similar to ALI patients, C57BL/6 mice that received an i.t. LPS developed a high level of neutrophils, increased pro-inflammatory cytokines, and inflicted tissue damage in the lung, which was suppressed by i.t. MGS administered at 2 h after LPS. Mechanistically, MGS activated Nrf2, which was related to MGS interrupting the ubiquitin-dependent degradation of Nrf2. MGS suppressed the nuclear localization of NF-κB induced by LPS, suggesting the inhibition of NF-κB activity. Furthermore, MGS inhibited the enzymatic activity of neutrophil elastase.

**Conclusion:**

MGS could suppress lung inflammation in an ALI mouse model, the effect of which could be attributed to multiple mechanisms, including the activation of Nrf2 and the suppression of NF-κB and neutrophil elastase enzymatic activity by MGS.

## Background

*Guettarda speciosa* is a perennial plant belonging to family Rubiaceae and found abundantly in tropical areas, such as the Okinawa Islands, Taiwan, tropical Asia, Australia, and the Polynesian Islands [1]. People in these areas have used *G. speciosa* to treat inflammatory diseases, including fever, colds, sore throats, fever, dysentery, and headache [2], suggesting that *G. speciosa* contains possibly anti-inflammatory activity. In support of this possibility, the methanol extract of *G. speciosa* Linn. (MGS) inhibits the expression of inducible NO synthase (iNOS) and IL-6 in RAW 264.7 cells [3]. Since iNOS and IL-6 are casually associated with inflammation [4], the study concluded that the anti-inflammatory activity of the herb is related to the suppression of these pro-inflammatory factors. Despite this reported mechanism, whether *G. speciosa* can suppress inflammation remains unclear because inflammation is not a reaction executed by a single type of cells. Rather, it involves complex, interrelated responses among migratory and residential cells at the histologic location where inflammation occurs. The complex nature of inflammation is well-documented in patients who suffer from acute lung injury (ALI), a medically unmet inflammatory lung disease [5, 6]. At the onset of inflammation, alveolar macrophages sense invaded bacteria via Toll-like receptor 4 (TLR4) [7, 8]. TLR4 signaling activated by LPS on the bacteria ends up the activation of NF-κB [9], resulting in the expression of cytokines such as TNF-α, IL-1β, IL-6, and IL-8 [6]. These cytokines promote inflammation by recruiting various inflammatory cells, notably neutrophils, to the lung [5]. Neutrophils infiltrated to the lung inflict damage to tissue by excreting various proteases, exacerbating inflammation [5]. Therefore, for the study of anti-inflammatory activities of medicinal herbs, it would be necessary to use an inflammatory disease mouse model.

Inflammatory reactions can be self-regulatory. For instance, activation of TLR4 in macrophages induces the production of reactive oxygen species (ROS) [4], resulting in the activation of nuclear erythroid 2-related factor 2 (Nrf2), an anti-inflammatory factor [10, 11]. The role of ROS is to inactivate Keap1. Keap1 functions as an inhibitor of Nrf2, in which Keap1 facilitates the ubiquitination of Nrf2 by linking Nrf2 and E3 ligase and thus promotes the ubiquitin-dependent degradation of Nrf2 [11, 12]. Thus, ROS inhibiting Keap1 prevents Nrf2 from ubiquitin-dependent degradation, resulting in an increased level of Nrf2. Since Nrf2 is a transcription factor, active Nrf2 enhances the expression of glutamate-cysteine ligase catalytic subunit (GCLC), NAD(P)H:quinine oxidoreductase-1 (NQO1), and heme oxygenase-1 (HO-1), which contribute to the suppression of inflammation [11, 12]. The important role of Nrf2 in ameliorating inflammatory diseases has been shown in various mouse models, including ALI and sepsis [13, 14]. Therefore, Nrf2 has been highlighted as a therapeutic target to treat ALI and other inflammatory diseases, along with NF-κB [15].

In this study, we investigated whether *G. speciosa* has anti-inflammatory activity by using an LPS-induced ALI mouse model. We fingerprinted the methanol extract of *G. speciosa* Linn. (MGS), and provide evidence that MGS can suppress inflammation in ALI mice. As for underlying mechanisms, we hypothesized that the anti-inflammatory function of MGS involves the activation of Nrf2 and the suppression of NF-κB, given that *G. speciosa* contains innumerable chemical constituents [16]. Here, we found the evidence supporting our hypothesis. Based on our results, we suggest that *G. speciosa* has anti-inflammatory activity.

## Methods

### Fingerprinting analysis of *Guettarda speciosa* Linn

The methanol extract of the stem and leaves of *Guettarda speciosa* Linn. (MGS; voucher #: FBM224–095) was obtained from the International Biological Material Research Center at the Korea Research Institute of Bioscience and Biotechnology, Daejeon, Korea. Fingerprinting MGS was conducted with Agilent 1200 series high-performance liquid chromatographic (HPLC) system. MGS (15 μl in methanol) was injected onto a column (CapcellPAK MGII C18, Shiseido, Japan, 4.6 × 150 mm ID, 3 μm). The column temperature was 35 °C, and the flow rate was 0.5 mL/min. Samples were eluted in a gradient of 0.1% formic acid and incremental acetonitrile in water. Standards and samples were detected at wavelengths of 254 ~ 320 nm. Index chemicals, including 3-*O*-caffeoylquinic acid, quercetin 3-*O*-galactoside, quercetin 3-*O*-glucoside, 3,4-di-*O*-caffeoylquinic acid, apigenin 7-*O*-glucuronide, and quercetin rutinoside, were purchased from Sigma-Aldrich (St. Louis, MO, USA). Chemstation software (Agilent Corporation, Germany) was used for data acquisition. The chemical constituents of MGS were identified by comparing their retention times to those of standard chemicals under identical analysis conditions and the UV spectra.

### MTT assay

Cytotoxicity was determined by using Vybrant® MTT assay kit and the manufacturer’s protocol (Thermo Scientific, IL, USA). Similar to the previous report [17], RAW 264.7 cells (ATCC, Rockville, MD, USA) were treated with MGS dissolved in PBS and incubated for 16 h in 5% CO_2_ incubator. Metabolically active cells were measured by a plate reader (BioTeK, VT, USA) and calculated against untreated cells to show in percentage. The assay was conducted in triplicate samples and repeated three times.

### Flow cytometry and FACS

Flow cytometric analysis was performed to measure intracellular ROS, as described elsewhere [17]. In short, RAW 264.7 cells (1 × 10^6^ cells/well) were incubated with 100 μM carboxy-H_2_DCFDA (Thermo Scientific) for 30 min and then analyzed with the BD FACS Canto II (BD Biosciences; San Jose, CA, USA). The expression of cytokines in inflammatory lung cells was similarly analyzed. After perfused with PBS, mouse lung was harvested, from which a single-cell suspension was prepared by using a lung dissociation kit (Miltenyi Biotech, Bergisch Gladbach, Germany). The suspended cells were incubated with anti-mouse IL-1β (NJTEN3), TNF-α (MP6-XT22), IL-6 (MP5-20F3), Ly-6G (1A8-Ly6g), and isotype control antibodies as recommended by the manufacturer (eBioscience, Waltham, MA, USA). Before adding those antibodies, Fcγ receptors were blocked by an anti-mouse CD16/32 antibody (2.4G2; BioLegend, San Diego, CA, USA). Unless specified otherwise, antibodies were incubated at 4 °C for 30 min. After being stained with propidium iodide, live cells were gated by forward scatter exclusion of dead cells. Data acquired by FACS Canto II were analyzed using FlowJo (Ashland, OR, USA).

### Acute lung injury (ALI) mouse model

Experiments with animals abided by the Guidelines for the Care and Use of Laboratory Animals (the NIH of Korea). The IACUC of Pusan National University approved the protocol of this study (protocol number: PNU-2016-1139). Male C57BL/6 mice (7 to 9 weeks old, Jackson Laboratory, Bar Harbor, ME, USA) were housed in certified, standard laboratory cages (3 to 5 mice per cage). Prior to the experiment, mice consumed the food and water ad libitum in a specific pathogen-free facility of Pusan National University. None of the C57BL/6 mice exhibited any distress and unexpected death while being housed. The detailed procedure was described elsewhere [18]. In brief, C57BL/6 mice (*n* = 5/group) anesthetized by Zoletil (Virbac, Carros Cedex, France), received a single intratracheal (i.t.) injection of 2 mg LPS/kg body weight (*E. coli* O55:B5, Sigma). Two hours later, mice received an additional i.t. injection of two different amounts of MGS that showed minimal cytotoxicity as determined by MTT assay in Fig. 2b. At 16 h after LPS, mice were sedated by Zoletil and subjected to bilateral bronchoalveolar lavage (BAL). Mice were injected with 1 mL of PBS via the trachea to the lung, which was repeated once more. In BAL fluid, we counted 300 cells in total from three different microscopic fields. The mean cell numbers of the three fields were shown in Fig. 3a and b. Using cell-free BAL fluid (2 mL) obtained after centrifugation, albumin was measured by ELISA (Abcam, Cambridge, UK; Fig. 3c). After euthanized by CO_2,_ mouse lungs were perfused with saline, inflated with fixatives, and then embedded in paraffin. Lung sections prepared in a 5 μm thickness were analyzed by hematoxylin and eosin (HE) staining [19]. At least three different lung slides per mouse were examined in 100X microscopic magnification.

### Western blot analysis

RAW 264.7 cells were treated with MGS or purified LPS (*Escherichia coli* O55:B5) specific to TLR4 (Alexis Biochemical, CA, USA). Nuclear proteins were prepared by NE-PER™ nuclear extraction kit per the manufacturer’s protocols (Thermo Scientific). The quantity of proteins was determined by Bradford (Bio-Rad, Hercules, CA, USA). Equal amounts of proteins were loaded and fractionated on NuPAGE gel (Thermo Scientific). Proteins were blotted to PVDF membrane (Bio-Rad). The blotted membranes were blocked with 5% non-fat dry milk for 1 h at room temperature (RT), incubated with primary antibodies at 4 °C overnight, and then with HRP-conjugated secondary antibodies (Santa Cruz Biotechnology, CA, USA) for 1 h at room temperature. Primary antibodies against Nrf2, p65 RelA, lamin B, and GAPDH were obtained from Santa Cruz Biotechnology, CA, USA. Proteins of interest were revealed by using SuperSignal®West Femto (Thermo Scientific).

### Ubiquitination assay

Ubiquitination assay was performed as described elsewhere [20]. In brief, HEK293 cells (ATCC) were transfected with plasmids expressing HA-tagged Ub, V5-tagged Nrf2, and FLAG-tagged Keap1 [21] for 48 h and then treated with MGS for 16 h. Prior to lysis, cells were treated with 10 μM of MG132 (Sigma-Aldrich) for 3 h to block ubiquitin-dependent protein degradation. The total cell lysate was prepared by Pierce™ IP lysis buffer per the manufacturer’s protocols (Thermo Scientific). For precipitating V5-tagged Nrf2, 1 μg of anti-V5 antibody (R960–25, Thermo Scientific) was added to the cytosolic fraction. Immune complexes captured by protein A-sepharose (Thermo Scientific) were analyzed by immunoblotting for HA (H3663, Sigma) to reveal the ubiquitinated Nrf2.

### Real-time quantitative PCR

Total RNA in RAW 264.7 cells was extracted by QIAGEN RNeasy®mini kit and the manufacturer’s protocol (Qiagen, Germany). One μg of the RNA was reverse-transcribed to cDNA by M-MLV reverse transcriptase (Promega, WI, USA), which was mixed with SYBR Green PCR Master Mix (Enzynomics, Daejeon, Korea) that contained gene-specific primers. The list of primers is as follows: the primers for NQO-1 were 5′-GCAG TGCTTTCCATCACCC-3′ and 5′-TGGAGTGTGCCCAATGCTAT-3′; those for HO-1 were 5′-TGAAGGAGGCCACCAAGGAGG-3′ and 5′-AGAGGTCACCCAGGTAGCG GG-3′; those for GCLC were 5′-CACTGCCAGAACACAGACCC-3′ and 5′-ATGGTCT GCTGAGAAGCCT-3′; and those for GAPDH were 5′-GGAGCCAAAAGGGTCATCA T-3′ and 5′-GTGATGGCATGGACTGTGGT-3′. The thermal reaction was run at 95 °C for 10 min, followed by 40 cycles of 95 °C for 10 s, 57 °C for 15 s, and 72 °C for 20 s in a Rotor-Gene Q real-time PCR system (Qiagen). The threshold cycles (Ct) were used to quantify the mRNA expression of the target genes.

### Neutrophil elastase assay

The effect of MGS on neutrophil elastase was determined by using the neutrophil elastase colorimetric drug discovery kit and the protocol provided by the manufacturer (Enzo Life Sciences, NY, USA). MGS diluted in PBS serially was mixed with human neutrophil elastase to be final 10 μg/mL, 1 μg/mL, 0.1 μg/mL, and 0.01 μg/mL. Elastase activities were determined by the absorbance at 405 nm, every minute for 10 min. Enzymatic activity in each sample was determined after plotting the reaction slope per the instruction of the manufacturer (Enzo Life Sciences).

### Statistical analysis

Paired or unpaired T-tests and one-way analysis of variance (ANOVA) tests were used (InStat, Graphpad Software, Inc., San Diego, CA). Data are shown in the mean ± SEM (Std. Error) of at least three measurements. *P* (≥0.05) was considered statistically significant.

## Results

### Fingerprinting of *Guettarda speciosa* Linn

**Fig. 1:**
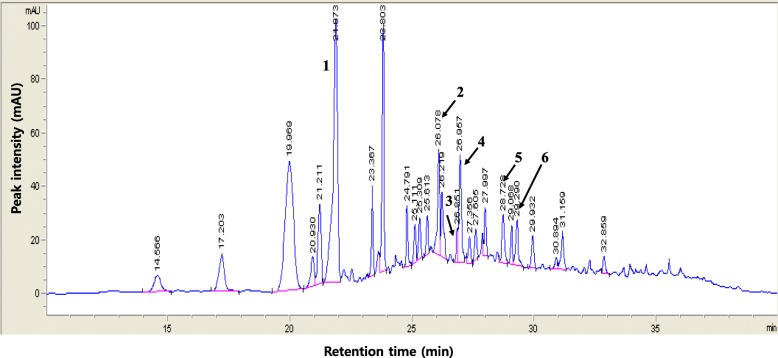
Fingerprinting of *Guettarda speciosa* Linn. The chemical constituents of the methanol extract of *G. speciosa* Linn. (MGS) was analyzed by HPLC. The compounds used as index chemicals are indicated as follow: 1, 3-*O*-caffeoylquinic acid (chlorogenic acid); 2, quercetin rutinosied (rutin); 3, quercetin 3-*O*-galactoside (hyperin); 4, quercetin 3-*O*-glucoside, (isoquercitrin); 5: 3,4-di-*O*-caffeoylquinic acid (isochlorogenic acid A); and 6, apigenin 7-*O*-glucuronide

The methanol extract of *Guettarda speciosa* Linn. (MGS) was fingerprinted by HPLC (Fig. 1). Given the report that MGS contains phenolic compounds, such as isoquercetin, chlorogenic acid, and isochlorogenic acid [16], they were included as index chemicals for MGS in this analysis. The constituents identified are indicated as follow: 1, chlorogenic acid (3-*O*-caffeoylquinic acid; CQA); 2, rutin (quercetin rutinosied; QRut); 3, hyperin (quercetin 3-*O*-galactoside; Q3 Gal); 4, isoquercitrin (quercetin 3-*O*-glucoside; Q3Glu); 5, isochlorogenic acid A (3,4-di-*O*-caffeoylquinic acid; DCQ); and 6, apigenin 7-*O*-glucuronide (A7Glu). The amounts of each chemical per 1 g of MGS dry weight were as follow: CQA, 4.13 mg/g; QRut, 2.09 mg/g; Q3Gal, 0.41 mg/g; Q3Glu, 1.70 mg/g; DCQ, 1.45 mg/g; and A7Glu, 2.01 mg/g.

### Cytotoxicity by MGS

**Fig. 2:**
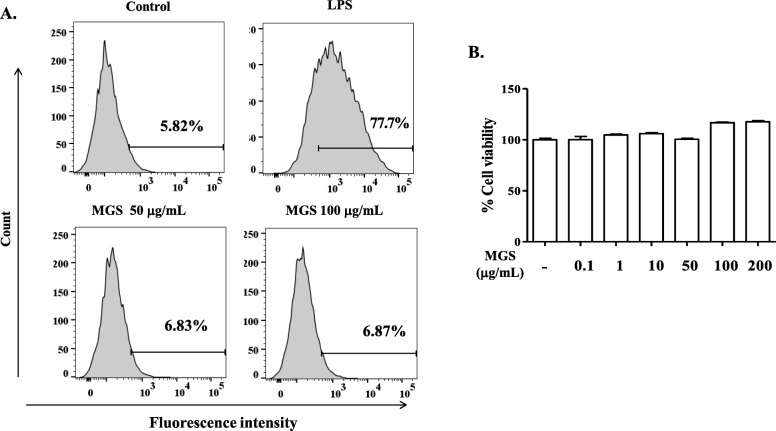
ROS production and cytotoxicity by MGS. (**a**) A flow cytometric analysis of intracellular ROS. After treated with LPS (100 ng/mL) or MGS (50 μg/mL and 100 μg/mL in PBS) for 16 h, RAW 264.7 cells were stained with carboxy-H_2_DCFDA, a ROS indicator. One thousand cells were counted, and cells producing intracellular ROS are shown in percentile, compared with untreated control. (**b**) MTT assay for cell viability. Increasing amounts of MGS were added to RAW 264.7 cells. After 16 h, metabolically active cells were determined after measuring the formazan product by a spectrophotometer. Data represent the mean ± SEM of triplicate samples. No statistical differences between groups were found (ANOVA)

Cytotoxicity could hamper the therapeutic usage of MGS. Thus, we examined possible cytotoxicity induced by MGS. As excessive reactive oxygen species (ROS) damage cells, which could cause cytotoxicity, we first tested if MGS increases ROS production (Fig. 2a). RAW 264.7 cells were treated with two high amounts of MGS (50 μg and 100 μg/mL), beyond which we considered ineffectual. At 16 h after treatment, flow cytometric analysis was performed to determine the level of intracellular ROS. As shown in Fig. 2a, unlike cells treated with LPS (top right panel), those treated with MGS produced ROS as low as the untreated control (the bottom two panels), suggesting that MGS induces no significant ROS production. Next, to test whether MGS induces cytotoxicity by interfering with cellular metabolism, we did an MTT assay. As shown in Fig. 2b, MGS up to 200 μg/mL did not significantly perturb the viability of RAW 264.7 cells, compared to the untreated control, suggesting no metabolic disarray caused by MGS. Together, these results suggest that MGS incurs no cytotoxicity.

### The effect of MGS on an acute lung injury in mice

**Fig. 3:**
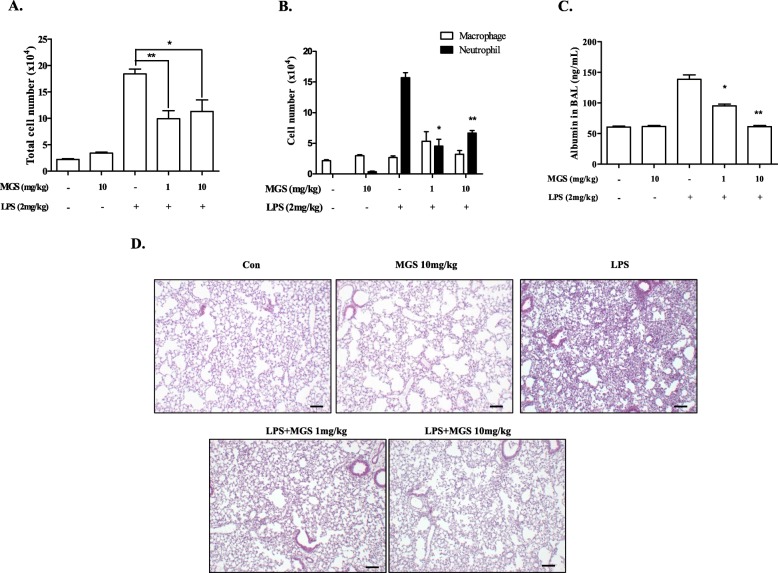
MGS decreases neutrophils and tissue damage in mouse lungs. C57BL/6 mice (*n* = 5 per group) were administered with a single i.t. PBS (control) or 2 mg/kg of i.t. LPS, along with MGS (1 mg/kg body weight or 10 mg/kg body weight). BAL was performed with PBS to obtain cells infiltrated to the lung. Total cells (**a**) and macrophages and neutrophils (**b**) in the BAL fluid were counted. From the cell-free BAL fluid, albumin was measured by using ELISA (**c)**. Data are shown in the mean ± SEM of three independent measurements. *P and **P were less than 0.001, compared to the LPS treated (post-ANOVA comparison with Tukey’s post hoc test). (**d**) Lung sections prepared were histologically analyzed after HE staining. At least three sections per mouse were analyzed under a microscope. Representative areas of each section are shown (100 magnifications, scale bar = 50 μm)

Next, we determined if MGS has anti-inflammatory activity by using an acute lung injury (ALI) mouse model, in which a high level of neutrophil infiltration to the lung and lung damage are featured [22]. Male mice (C57BL/6) were randomly grouped (*n* = 5/group). Two groups were treated with a single administration of i.t. PBS (control and MGS groups) and the other three groups were with i.t. LPS (2 mg/kg body weight) to induce ALI. Two hours later, PBS- or LPS-treated mice received a single i.t. of PBS or MGS (LPS with or without MGS groups). At 16 h after LPS treatment, mice were sacrificed for the analysis of lung tissue and inflammatory cells in the lung (Fig. 3). Bronchoalveolar lavage (BAL) was performed with PBS, and the cells in the BAL fluid were analyzed under the microscope. As shown in Fig. 3a, LPS induced a high degree of cellular infiltration to the lung, which was significantly suppressed by MGS treatment (3rd, 4th, and 5th columns). Differential cell counting (Fig. 3b) shows that LPS substantially increased the level of neutrophils in the lung, an archetypic characteristic of the inflamed lung in ALI [22], which was significantly suppressed by MGS. This effect was evident when mice received 1 mg/kg body weight of MGS (3rd and 4th filled columns). These results suggest that MGS suppresses neutrophilic lung inflammation.

Since tissue damage comes along in ALI [23], we determined whether MGS also decreases tissue damage. Since tissue damage leads to blood leakage into the airspace in the lung, we measured the level of blood albumin as a surrogate of tissue destruction. A cell-free BAL fluid was prepared, in which albumin was measured by ELISA. As shown in Fig. 3c, while LPS increased the level of albumin in the BAL fluid (3rd column), indicating lung tissue damage by LPS, MGS significantly suppressed it (4th and 5th columns). Consistent with these, HE staining of lung sections (Fig. 3d) shows that LPS increased cellular infiltration and the formation of some hyaline membrane, a histologic characteristic of the inflamed lung (top right panel), which was substantially ameliorated by MGS (bottom two panels).

**Fig. 4:**
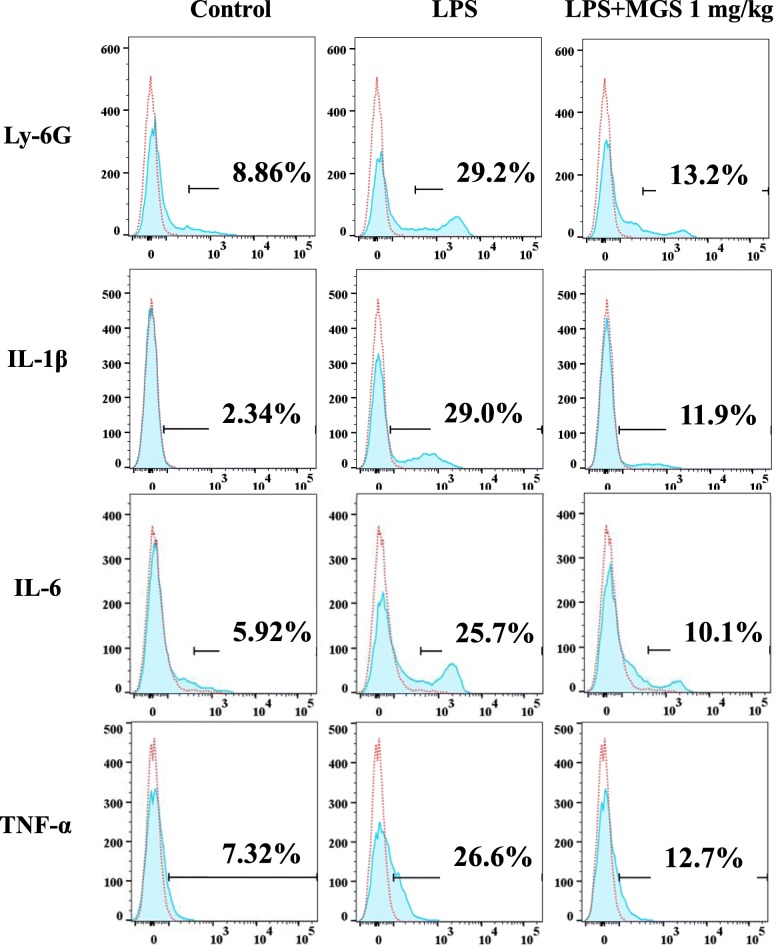
MGS decreases the number of cells expressing inflammatory cytokines. From C57BL/6 mice (*n* = 3/group) treated with i.t. PBS (control), i.t. LPS (2 mg/kg body weight), or LPS with i.t. MGS (1 mg/kg body weight), single-cell suspension of the lung was prepared and incubated with anti-Ly-6G, −TNF-α, −IL-1β, or -IL-6 antibodies. Representative histograms after flow cytometric analyses are shown. The percentile of positive cells is denoted in each panel

As inflammatory cytokines, including TNF-α, IL-1β, and IL-6, trigger cell infiltration to the lung [4], we analyzed cells in the lung tissue. A single-cell suspension was prepared from the whole lung after digested with collagenase D [24] and analyzed by FACS. First, we determined whether MGS suppresses neutrophil infiltration. As shown in Fig. 4, while LPS increased the level of Ly-6G^+^ cells (neutrophils; top center panel), MGS decreased the level of cells from 29.3 to 13.2% (top right panel). This result was consistent with those in Fig. 3b, suggesting that MGS dampens neutrophil infiltration induced by LPS. Next, we scored cells positive with TNF-α, IL-1β, and IL-6 to determine whether MGS suppresses the expression of inflammatory cytokines. As shown in panels in Fig. 4, MGS abated the levels of cells positive with each cytokine (right panels) that were increased by LPS (middle panels). Combined, these results suggest that MGS suppresses neutrophilic inflammation, along with decreased cytokine production.

### MGS activates an anti-inflammatory factor Nrf2

**Fig. 5:**
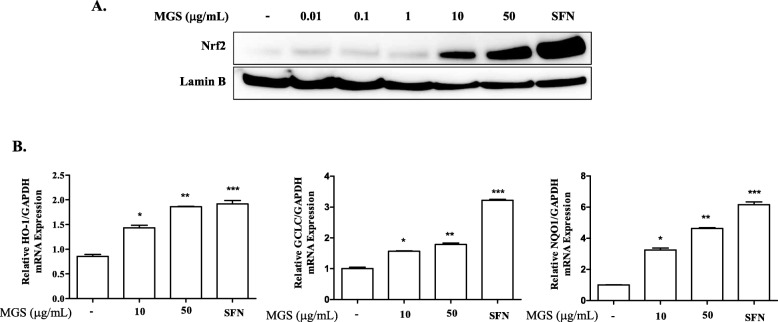
MGS activates Nrf2. (**a**) RAW 264.7 cells incubated with the indicated amounts of MGS or sulforaphane (5 μM) for 16 h were harvested. Nuclear fraction was analyzed by immunoblotting for Nrf2. The same blot was stripped and incubated with anti-lamin B antibody. A similar experiment was performed three times. (**b**) Total RNA extracted from RAW 264.7 cells treated with MGS was analyzed for the expression of GCLC, HO-1, and NQO-1 by a quantitative real-time PCR. Data represent the mean ± SEM of three independent measurements. *P, **P, and **P were less than 0.001, compared to untreated controls

Given the anti-inflammatory activity of MGS found in the study, we explored possible underlying mechanisms. Since Nrf2 is a critical anti-inflammatory factor, we first examined if MGS activates Nrf2, contributing to the anti-inflammatory activity of MGS. Varying amounts of MGS were added to RAW 264.7 cells for 16 h. Nuclear proteins were isolated and analyzed by immunoblotting for nuclear Nrf2, an active form of Nrf2 [11]. As shown in Fig. 5a, MGS increased the level of the nuclear Nrf2, which became evident when cells were treated with 10 μg/mL of MGS, compared to those with sulforaphane (5 μM), an Nrf2 activator [25]. Since Nrf2 is a transcription factor, we examined whether MGS increases the Nrf2-dependent gene expression. After RAW 264.7 cells were treated with MGS similarly to Fig. 5a, total RNA was extracted from the cells and then analyzed by a quantitative real-time PCR (qPCR) to measure the expression of prototypic Nrf2-dependent genes, such as HO-1, GCLC, and NQO-1 [11, 12]. As shown in Fig. 5b, MGS induced the expression of these genes. Together, these results suggest that MGS activates Nrf2.

**Fig. 6:**
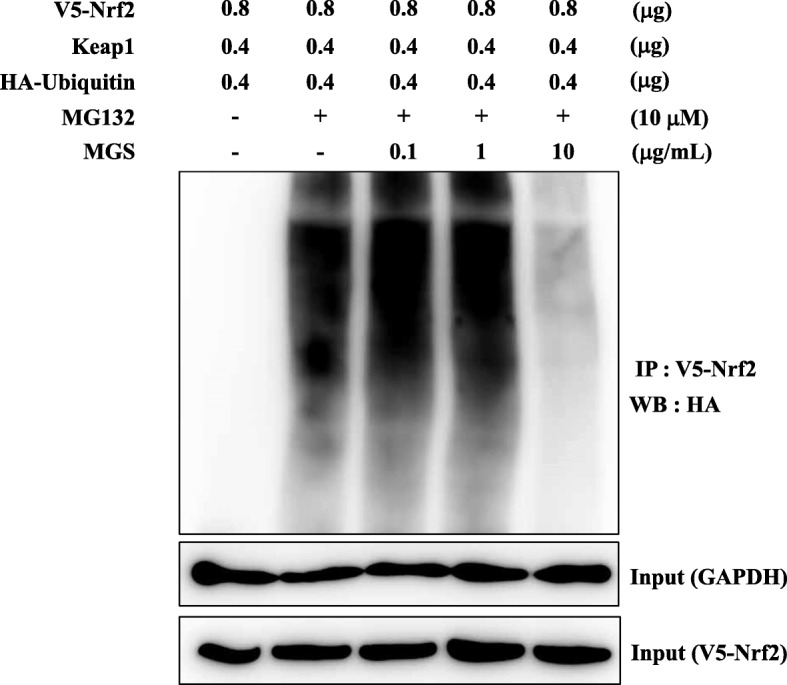
MGS suppresses the ubiquitination of Nrf2. HEK 293 cells transfected with plasmids encoding V5-Nrf2, HA-Ub, and Keap1 were treated with MGS for 16 h. After V5-Nrf2 was immunoprecipitated by an anti-V5 antibody, the ubiquitinated V5-Nrf2 was analyzed by an anti-HA antibody. The cell lysate was analyzed by immunoblotting for V5-Nrf2 and GAPDH as for inputs

To understand how MGS activates Nrf2, we tested the possibility that MGS interrupts the ubiquitination of Nrf2, increasing the level of the nuclear Nrf2. To address this, we co-transfected HEK 293 cells with the plasmids encoding V5-Nrf2, FLAG-Keap1, and HA-ubiquitin for 48 h. Transfected cells were subsequently treated with MGS for 16 h. Three hours prior to cell harvest, MG 132, a proteasome inhibitor that blocks the ubiquitin-dependent degradation of proteins [26, 27], was added to the cells. V5-Nrf2 was precipitated with an anti-V5 antibody, and the degree of ubiquitination in V5-Nrf2 was assessed by immunoblotting for HA (HA-ubiquitin). As shown in Fig. 6, while strongly ubiquitinated in the presence of Keap1 (2nd lane), Nrf2 was readily degraded in the absence of MG 132 (1st lane). However, 10 μg/mL of MGS suppressed the ubiquitination of Nrf2 (5th lane). These results suggest that MGS interferes with the ubiquitination and thus the degradation of Nrf2, contributing to the increase of the nuclear Nrf2.

### MGS suppresses a pro-inflammatory factor NF-κB and neutrophil elastase activities

**Fig. 7:**
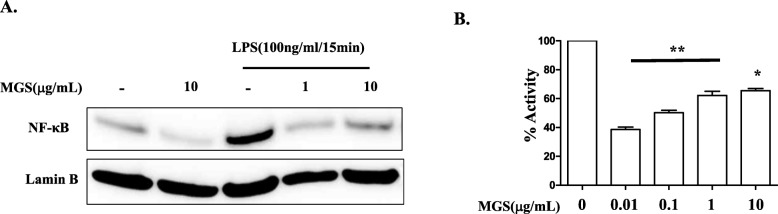
MGS suppresses NF-κB and neutrophil elastase activity. (**a**) RAW 264.7 cells were treated with MGS for 16 h and subsequently with 100 ng/mL of LPS for 15 min. Nuclear proteins were analyzed by immunoblotting for p65 RelA. The same blot was stripped and reblotted for lamin B. Similar experiments were performed three times. (**b**) MGS diluted in PBS was added to neutrophil elastase at final concentrations of 0.01, 0.1, 1, 10 μg/mL. Elastase activity was measured every minute for 10 min by ELISA assay per the instruction of the manufacturer. Data represent the mean ± SEM of three independent measurements. *P and **P were less than 0.01 and 0.001, respectively, compared to the control elastase activity

Since MGS suppressed the expression of TNF-α, IL-1β, and IL-6, we examined whether MGS suppresses NF-κB activity, a transcription factor that regulates the expression of those pro-inflammatory cytokines [16, 28]. RAW 264.7 cells were treated with MGS for 16 h and subsequently with 100 ng/mL of LPS for 15 min. Nuclear proteins were extracted and analyzed by immunoblotting for nuclear p65 RelA, indicative of NF-κB activated [29]. As shown in Fig. 7a, LPS that activates NF-κB [28] increased the level of the nuclear p65 RelA (lane 3), which was decreased by MGS treatment (lanes 4 and 5), suggesting that MGS suppresses the activity of NF-κB.

As MGS suppressed lung tissue damage, we examined the possibility that MGS inhibits the activity of proteases secreted by neutrophils, which contributes to the destruction of lung tissue [30]. MGS was serially diluted in PBS and added to neutrophil elastase to be final 10□μg/mL to 0.01 μg/mL. The effect of MGS on neutrophil elastase activity was determined by ELISA. As shown in Fig. 7b, a high amount of MGS, 10 μg/mL, suppressed the elastase activity down to about 65%, compared to the control elastase activity, to which no inhibitor was added. The lowest amount of MGS used in the experiment, 0.01 μg/mL, suppressed the elastase activity down to about 38%, suggesting the low amount of MGS is more effective in suppressing elastase activity. Nevertheless, these results suggest that MGS suppresses elastase activity.

## Discussion

Notwithstanding the traditional use of *G. speciosa* to treat inflammatory diseases, studies to support the anti-inflammatory effect of *G. speciosa* are scarce. The purpose of this study was to obtain experimental evidence that *G. speciosa* had anti-inflammatory activity, along with a glimpse at possible underlying mechanisms. With the methanol extract of *G. speciosa* Linn. (MGS), we presented the evidence that *G. speciosa* contained anti-inflammatory activity. Our results show that when delivered to mouse lung via an intratracheal (i.t.) route, MGS suppressed neutrophilic infiltration to the lung and reduced tissue damage incurred by i.t. LPS. Accordantly, MGS decreased the levels of cells that expressed prototypic pro-inflammatory cytokines in mouse lungs. With RAW 264.7 cells, we show that MGS activated an anti-inflammatory factor Nrf2 and induced the expression of Nrf2-dependent genes such as HO-1, GCLC, and NQO-1. MGS suppressed the ubiquitination of Nrf2, suggesting that MGS interferes with ubiquitin-dependent degradation of Nrf2, which could account for Nrf2 activated by MGS. Furthermore, we evidenced that the anti-inflammatory function of MGS involved the suppression of pro-inflammatory factors NF-κB and of neutrophil elastase activity. Together, our findings suggest that MGS has the capability of suppressing neutrophilic inflammation, with a multitude of mechanisms involving the activation of Nrf2, the suppression of NF-κB, and the inhibition of neutrophil elastase activity.

Since we have looked for herbal medicine effectual in inflammatory lung diseases, we tested whether MGS can suppress inflammation in a mouse model of ALI, a rampant inflammatory lung disease with high mortality [5]. In our experimental setting, LPS instilled into the mouse lung closely recapitulated the hallmarks of ALI, including neutrophil infiltration to the lung, tissue damage, increased expression of pro-inflammatory cytokines, and histologic changes with the hyaline membrane [31]. In our experiment, it was apparent that MGS suppressed the infiltration of neutrophil to the lung, which was confirmed by Ly-6G^+^ cells, mostly neutrophils [32], decreased by MGS. In addition, MGS reduced the populations of TNF-α^+^, IL-1β^+^, and IL-6^+^ cells and ameliorated tissue damage, suggesting that MGS suppresses lung inflammation in our ALI mouse model. We noticed that the percentage of cells expressing the pro-inflammatory cytokines was not high, considering that these cytokines are, in general, produced robustly shortly after an LPS challenge [33]. The relatively weak expression of these pro-inflammatory cytokines could be due to that they were measured at 16 h after i.t. LPS treatment. Although MGS could suppress the increase of inflammatory cells positive with these cytokines at the early stage of inflammation, it is not clear at this moment how effectively did in an early stage of inflammation. Nonetheless, our findings show that MGS decreased the expression of these cytokines along with ameliorating other characteristics found in ALI, suggesting the possibility that MGS helps tip the scale of inflammation toward resolution.

Deciphering the mechanisms underlying the anti-inflammatory activity of MGS would be challenging. One of the reasons is that *G. speciosa* is composed of numerous chemical constituents [2]. Given inflammatory reaction involves various cell types and complex interactions among them [34], it is highly likely that MGS affects multiple pathways in cells that are engaged in the inflammatory reaction. A recent study shows that MGS inhibits the expression of inducible NO synthase (iNOS) and IL-6 in RAW 264.7 cells treated with lipopolysaccharide (LPS) [3]. Since MGS suppresses the activities of spleen tyrosine kinase (Syk) and c-Jun N-terminal kinase (JNK) [3], the study suggested that suppression of these kinases accounts for the anti-inflammatory effect of MGS. Our study proposes other possible mechanisms. We show that MGS induced the nuclear localization of Nrf2, indicative of activated Nrf2, and the increase of mRNAs of GCLC, NQO-1, and HO-1, representative Nrf2-dependent genes [27]. Our results show that MGS suppressed the ubiquitination of Nrf2, suggesting that MGS activating Nrf2 is related to interrupting the ubiquitination of Nrf2. This possibility is consistent with the finding that Nrf2 is largely down-regulated via ubiquitin-dependent degradation [35]. It is of note that MGS did not induce the production of ROS. As ROS inactivate Keap1, resulting in the activation of Nrf2 [36], our results suggest that MGS directly interferes with the ubiquitin-dependent degradation of Nrf2 without ROS involved. Furthermore, a plethora of studies have shown that Nrf2 plays a role in ameliorating pulmonary inflammatory diseases, including ALI, emphysema, and asthma [37–40]. Therefore, it is conceivable that MGS activating Nrf2 contributes at least in part to the anti-inflammatory activity of MGS.

MGS decreased the numbers of cells producing pro-inflammatory cytokines such as TNF-α, IL-1β, and IL-6 in mouse lungs. Since the expression of these cytokines is regulated by NF-κB, a transcription factor that promotes inflammatory reaction [29], we tested the possibility that MGS suppresses the activity of NF-κB. In fact, the crucial role of NF-κB in regulating inflammation has been well-documented throughout numerous studies and diseases models [28]. As shown in our study, MGS suppressed the nuclear localization of NF-κB induced by LPS, suggesting MGS suppressing NF-κB. In conjunction with the results of Nrf2, these results suggest that the anti-inflammatory activity of MGS is attributable to not only activating Nrf2 but suppressing NF-κB.

Inflammatory cytokines promote inflammatory reactions, in part, by recruiting neutrophils to the affected lesion. Neutrophils recruited engulf and thus remove invading bacteria via phagocytosis. Neutrophils also excrete various proteases, which help enhance the anti-microbial function of phagocytic cells [41]. Although these enzymes are critical in destroying pathogens, they collaterally inflict damage to tissue, exacerbating inflammation [41, 42]. Our results show that MGS decreased lung tissue damage. While this effect could be due to decreased neutrophil recruitment, MGS might inhibit the activity of neutrophil elastase, a protease that is involved in ALI [42]. Indeed, our results show that MGS inhibited the enzymatic activity of elastase at as low as 0.01 μg/mL. The inhibitory effect of MGS was the highest at the lowest amount tested in this experiment, suggesting that MGS inhibits the elastase activity with relatively high specificity. As neutrophils excrete numerous proteases along with elastase, it would be interesting to examine whether MGS could suppress the activities of other related enzymes.

## Conclusions

MGS suppressed neutrophilic lung inflammation in an LPS-induced ALI mouse model. The likely mechanisms by which MGS exerts the anti-inflammatory effect involve the activation of an anti-inflammatory factor Nrf2, the suppression of a pro-inflammatory factor NF-κB, and the inhibition of neutrophil elastase. We propose that MGS has anti-inflammatory activity and is applicable to treat acute inflammatory diseases, including ALI.

## Data Availability

The data sets and materials are readily available upon request to the corresponding authors.

## Abbreviations

ALI: Acute lung jury
HPLC: High-performance liquid chromatography
IL-1β: Interleukin-1β
IL-6: Interleukin-6
IL-8: Interleukin-8
LPS: Lipopolysaccharide
MGS: The methanol extract of *Guettarda speciosa* Linn
MTT: 3-(4,5-dimethylthiazol-2-yl)-2,5-diphenyltetrazolium bromide
NF-κB: Nuclear factor kappa-light-chain-enhancer of activated B cells
Nrf2: Nuclear erythroid 2-related factor 2
qPCR: Quantitative polymerase chain reaction
TNF-α: Tumor necrosis factor-α
